# Evaluation of In Vitro Biological Activity of Flavanone/Chromanone Derivatives: Molecular Analysis of Anticancer Mechanisms in Colorectal Cancer

**DOI:** 10.3390/ijms252312985

**Published:** 2024-12-03

**Authors:** Pawel Hikisz, Piotr Wawrzyniak, Angelika A. Adamus-Grabicka, Damian Jacenik, Elzbieta Budzisz

**Affiliations:** 1 Department of Oncobiology and Epigenetics, Institute of Biophysics, Faculty of Biology and Environmental Protection, University of Lodz, Pomorska 141/143, 90-236 Lodz, Poland; 2 Department of Molecular Biology of Cancer, Medical University of Lodz, 6/8 Mazowiecka Street, 92-215 Lodz, Poland; piotr.wawrzyniak@student.umed.lodz.pl; 3 Department of Bioinorganic Chemistry, Faculty of Pharmacy, Medical University of Lodz, Muszynskiego 1, 90-151 Lodz, Poland; angelika.adamus@umed.lodz.pl; 4 Department of Cytobiochemistry, Faculty of Biology and Environmental Protection, University of Lodz, ul. Pomorska 141/143, 90-236 Lodz, Poland; damian.jacenik@biol.uni.lodz.pl; 5 Department of the Chemistry of Cosmetic Raw Materials, Medical University of Lodz, 90-151 Lodz, Poland; elzbieta.budzisz@umed.lodz.pl

**Keywords:** flavonoid derivatives, anticancer properties, flavanone, chromanone, apoptosis, reactive oxygen species, genotoxicity, colon cancer, structure–activity relationship

## Abstract

The primary objective of this study was to evaluate the anticancer activity of six flavanone/chromanone derivatives: *3*-benzylideneflavanones/*3*-benzylidenechroman-4-ones and their *3*-spiro-*1*-pirazolines analogs. We employed five colon cancer cell lines with varying degrees of metastasis and genetic profiles as our research model. Our investigation focused primarily on assessing the pro-oxidant properties of the tested derivatives and their impact on overall antiproliferative activity. To comprehensively evaluate the cytotoxic properties of these compounds, we analyzed their genotoxic, pro-apoptotic, and autophagy-inducing effects. Our findings indicate that three of the six analyzed derivatives exhibited promising antiproliferative activity against cancer cells, with IC_50_ values ranging from 10 to 30 μM. Strong pro-oxidant properties were identified as a key mechanism underlying their cytotoxic activity. The generation of oxidative stress, which varied depending on the specific flavanone/chromanone derivative, resulted from increased intracellular reactive oxygen species (ROS) levels and decreased glutathione (GSH) concentrations. Furthermore, oxidative stress likely contributed to the induction of apoptosis/autophagy in cancer cells and the emergence of significant DNA damage.

## 1. Introduction

Cancer, alongside circulatory system diseases, is one of the most significant causes of human mortality worldwide [1]. This is undoubtedly a negative consequence of humanity’s rapid civilizational and technological advancement. While industrialization and lifestyle changes have increased some risks, advancements in medicine and technology have also improved life expectancy and treatment outcomes. Despite substantial progress in medical and pharmacological knowledge and the widespread availability of various preventive programs, the battle against cancer is often lost. The phenomenon of increased life expectancy and the progressive aging of populations in highly and moderately developed countries exacerbates the cancer problem [1,2].

Colorectal cancer is one of the major global health concerns. Numerous studies indicate that it is a leading cause of cancer-related deaths, and the overall incidence of colorectal cancer continues to rise [3]. While previously more prevalent in developed countries, this type of cancer is now increasingly affecting developing nations. The etiology of colorectal cancer is complex and not fully understood, despite ongoing medical advancements. It is considered a multi-stage and intricate process influenced by a wide range of environmental and genetic factors. A significant proportion of colorectal cancer cases are sporadic, arising from lifestyle, dietary, and nutritional factors. Familial genetic predisposition accounts for less than 30% of cases [3,4].

Epidemiological studies have consistently identified environmental factors as major risk factors. An unhealthy lifestyle characterized by excessive alcohol consumption, tobacco use, a diet high in empty calories and low in nutrients, chronic stress, physical inactivity, antibiotic overuse, smoking, and obesity can contribute to an increased risk of colorectal cancer. These factors can lead to metabolic dysfunctions, inflammation, oxidative stress, disruption of the intestinal microbiota, and impaired intestinal barrier integrity [5,6].

While environmental factors play a significant role, genetic factors are also important in the pathogenesis of colorectal cancer. Hereditary or spontaneous mutations in specific genes, combined with environmental factors, can initiate carcinogenesis. Most colorectal cancer cases arise from de novo mutations acquired during a person’s lifetime. However, some individuals are predisposed to the disease due to inherited germline mutations in genes such as Adenomatous Polyposis Coli (*APC*), Kirsten rat sarcoma virus (*KRAS*), and tumor protein p53 (*TP53*), which significantly increase their risk [7].

The search, synthesis, and achievement of appropriate physicochemical stability of new compounds with desired pharmacological activity remain a significant challenge for modern medicine. This is particularly crucial in light of the increasing prevalence of multi-drug resistance in cancers, which limits the efficacy of current chemotherapeutics. While conventional chemotherapeutics exhibit satisfactory anticancer activity, they are often associated with substantial systemic cytotoxicity, hindering their widespread use. The lack of specificity of commercially available chemotherapeutics has driven increased interest in novel derivatives with promising anticancer properties and reduced systemic toxicity [5,6,8].

Flavonoids, a diverse group of compounds classified into six categories (isoflavonoids, flavanones, flavanols, flavonols, flavones, and anthocyanidins), have long been a subject of scientific interest due to their broad spectrum of biological activities. Their potential application in cancer therapy is particularly intriguing. While the precise mechanism of their anticancer action remains elusive, recent studies increasingly suggest that flavonoids can serve as a valuable complement or alternative to conventional treatments [8].

Flavonoids can influence a wide array of biological molecular pathways and processes, thereby exerting their anticancer and antiproliferative effects. These compounds can modulate the activity of enzymes involved in reactive oxygen species scavenging, induce cell cycle arrest, and trigger apoptosis and autophagy [9].

Heterocyclic compounds derived from natural sources have emerged as promising candidates for the development of anticancer therapies. Both their native forms and chemically modified derivatives have shown significant potential. A particularly effective approach in the discovery of novel anticancer agents involves the chemical modification of naturally occurring substances with established anticancer properties. The importance of heterocyclic rings in drug design is highlighted by the fact that over 65% of FDA-approved anticancer drugs between 2010 and 2015 incorporated such structures [10].

For many years, our research team has focused on synthesizing biologically active compounds containing heterocyclic rings, specifically *3*-benzylideneflavanones/*3*-benzylidenechroman-4-ones with various substituents attached in the ortho, meta, or para positions and their *3*-spiro-*1*-pirazolines, and investigating their molecular mechanisms of anticancer activity. Extensive research has demonstrated the promising anticancer properties of these compounds, which exhibit antiproliferative and cytotoxic effects through various molecular pathways against diverse cancer types [11,12,13].

**Scheme 1:**
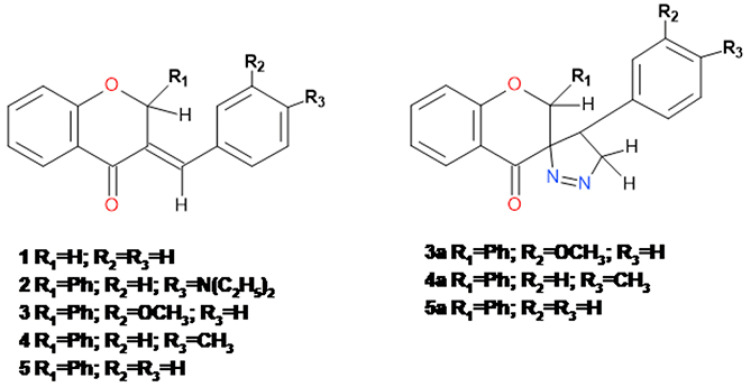
The structures of tested compounds.

In our previous studies, we comprehensively described the biological activity, physicochemical properties, and synthesis of *3*-spiro-*1*-pirazolines condensed with flavanone or chromanone derivatives [14]. In this work, we selected six compounds (flavanone/chromanone derivatives) (**1**, **2**, **3**, **3a**, **4**, and **5**, as depicted in Scheme 1) to evaluate their anticancer activity and analyze the underlying molecular mechanisms responsible for inhibiting cancer cell proliferation.

## 2. Results and Discussion

### 2.1. Evaluation of Cell Viability by MTT Spectrophotometric Method 

We investigated the molecular anticancer mechanisms of titled compounds against five human colon cancer cell lines: HCT 116, SW620, LoVo, Caco-2, and HT-29. These compounds exhibit pleiotropic effects, targeting multiple cellular pathways, and often have lower toxicity profiles than traditional therapies. We selected these colon cancer cell lines to establish a comprehensive model for investigating the therapeutic potential of flavanone/chromanone derivatives against colon cancer. The chosen cell lines are well characterized genetically and molecularly, enabling robust comparisons across studies. Furthermore, we aimed to represent the diverse genetic and molecular heterogeneity of colon cancer by including cell lines from various subtypes.

The cytotoxic activity of the *3*-benzylideneflavanones, *3*-benzylidenechromanones derivatives (compounds **1**, **2**, **3**, **4**, **5**) and 5′-(3-methoxyphenyl)-2-phenyl-4′,5′-dihydro-4*H*-spiro[chromano-3,3′-pirazol]-4-one (compound **3a**) was evaluated using the MTT assay (4,5-dimethylthiazol-2-yl)-2,5-diphenyltetrazoliumbromide), a colorimetric method that quantifies cell viability. Based on the cell survival curves generated for each flavanone/chromanone derivative, the IC_50_ value was determined, representing the concentration required to inhibit cell proliferation by 50% compared to control cells. These IC_50_ values were subsequently used for all experiments investigating the anticancer activity of the flavanone/chromanone derivatives in this study. The calculated IC_50_ values are presented in Table 1.

Based on these results, the compounds were categorized into two groups: those exhibiting potent antiproliferative activity against colon cancer cell lines and those displaying significantly weaker anticancer effects. Notably, in most cases, the tested compounds demonstrated a pronounced polarization of biological activity: either they exhibited comparable or even slightly better antiproliferative potency than the reference compound (cisplatin), or their activity was consistently low. A consistent pattern of biological activity was observed for all compounds. The exceptions here are the HT-29 and Caco-2 lines, which were primarily sensitive to derivatives **1** and **5** for HT-29 and derivative **1** for Caco-2, respectively. It is worth noting that obtaining an appropriately high selectivity of cytotoxic activity of potential chemotherapeutics is still a major challenge. The MTT cytotoxicity results that we obtained for the tested flavanone/chromanone derivatives confirm this scientific problem. The calculated IC_50_ values for the normal HMEC-1 line were comparable to those obtained for the majority of tested cancer cell lines. Notably, the most potent anticancer compound, derivative **1**, demonstrated superior selectivity. Its IC_50_ value against HMEC-1 cells was approximately 10 µM higher than that observed for the majority of tested cancer cell lines.

Among the six compounds evaluated, three exhibited negligible antiproliferative activity against all cell lines. Compounds **2**, **3a**, and **4** displayed cytotoxic effects on cancer cells within an IC_50_ range of approximately 70–210 µM. In contrast, the remaining three compounds, **1**, **3**, and **5**, demonstrated promising biological properties. For most cell lines, the IC_50_ values of these derivatives were within the range of ~8–30 µM. Among all derivatives, compound **1** exhibited the most potent antiproliferative activity. This compound demonstrated a broad spectrum of biological activity by effectively inhibiting the proliferation of all five cancer cell lines. The IC_50_ concentration of derivative **1** was within the low micromolar range of 8–20 µM, comparable to the reference compound cisplatin. Compounds **3** and **5** displayed slightly weaker antiproliferative effects, with IC_50_ values ranging from 15 to 30 µM depending on the cancer cell line. Notably, the Caco-2 and HT-29 cell lines exhibited the lowest sensitivity to the flavanone/chromanone derivatives. Derivative **1** was the only compound to effectively inhibit Caco-2 cell proliferation at low micromolar concentrations. While HT-29 cells were sensitive to compounds **1** and **5**, the IC_50_ values for these compounds were nevertheless approximately 10 µM higher compared to the other cell lines.

The observed results suggest that the overall anticancer activity of flavanone derivatives is influenced by structure–activity relationships (SARs). Structural modifications can alter the ability of these compounds to interact with cellular targets, thereby impacting their biological activity. An analysis of the antiproliferative activity of the six flavanone/chromanone derivatives reveals distinct structure–activity relationships. They have a very specific three-ring structure: two phenols (rings **A** and **B**) joined by a pyran or oxygen-containing hexagonal (ring **C**) structure. Specific modifications to the flavonoid skeleton, particularly at the C3 carbon of the C ring, significantly influence the overall anticancer activity against the tested cell lines. The attachment of a benzene ring via a double π bond at C3, as observed in compounds **1**, **3**, and **5**, is associated with enhanced antiproliferative potency. Conversely, the presence of the *para-* or *ortho*- methylphenyl-4,5-dihydro-3H-pyrazole ring at C3 significantly attenuates antiproliferative activity. The pair of compounds **3** and **3a** exemplifies this relationship, with compound **3** (lacking the five-membered pyrazole ring) demonstrating superior biological activity and compound **3a,** containing the mentioned pyrazole ring in its structure, remaining biologically active to a very small extent. Furthermore, analysis of the structures of the tested derivatives and their cytotoxic activity suggests that the R3 substituents on the phenol B ring significantly influence the overall antiproliferative potential of a given molecule. Notably, derivatives 2 and 4, bearing N(C_2_H_5_)_2_ and CH_3_ groups at the R3 position, respectively, exhibited reduced cytotoxicity compared to derivatives 1 and 5, which lacked an R3 substituent. Based on the results presented in this study, including the significant generation of reactive oxygen species (ROS), it is plausible to hypothesize that the π double bond systems within flavanone/chromanone derivatives play a pivotal role in determining their potent antiproliferative and anticancer activities, which will be discussed in detail in Section 2.3.

Based on cytotoxicity results and IC_50_ values, we selected the most potent flavanone/chromanone derivatives for further investigation. Compounds **1**, **3**, and **5** were chosen due to their promising antiproliferative activity in preliminary screening for further studies planned in this work. An IC_50_ threshold of 35 µM was established as a cutoff for considering compounds as potentially biologically active chemotherapeutic candidates. For the Caco-2 line, only derivative **1** met the aforementioned criteria, while derivatives **3** and **5** exhibited high, unsatisfactory IC_50_ values of approximately 70 µM. Consequently, only compound **1** was selected for further studies with this cell line. Similarly, for the HT-29 line, compounds **1** and **5** demonstrated satisfactory IC_50_ concentrations, whereas derivative **3** exhibited negligible cytotoxic activity (IC_50_ ≈ 115 µM) and was therefore excluded from subsequent investigations.

### 2.2. Measurement of the Kinetics of Mitochondrial Potential Changes (ΔΨm) Using a Microplate Spectrofluorimetric Method with a Fluorescent Probe JC-1

Mitochondrial membrane potential (ΔΨm) is crucial for cellular homeostasis and ATP production via oxidative phosphorylation. Alterations in ΔΨm, including hyperpolarization and depolarization, can trigger cellular responses, notably apoptosis. Initial apoptotic events involve ΔΨm hyperpolarization, followed by irreversible depolarization, mPTP opening, and release of pro-apoptotic factors like cytochrome c. Depolarization also activates pro-apoptotic Bcl-2 family members, such as Bax and Bak [15,16].

**Figure 1:**
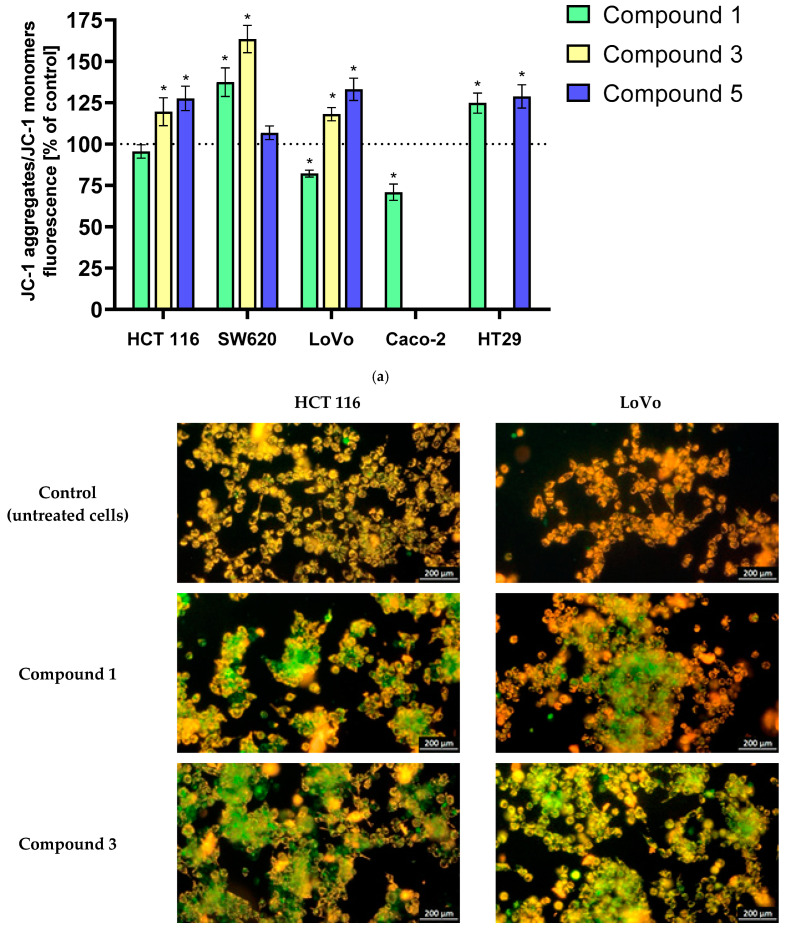

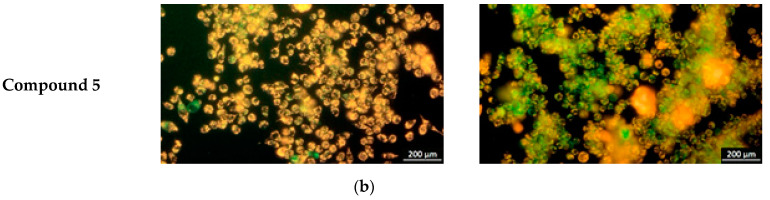
(**a**). Analysis of changes in the mitochondrial membrane potential (MMP) of HCT 116, SW620, LoVo, Caco-2, and HT-29 colon cancer cells treated with the investigated flavanone/chromanone derivatives (IC_50_ concentrations) using the fluorescent probe JC-1. The results are expressed as a percentage of the control, calculated by comparing the fluorescence intensity ratio of the test sample to the control (100%). Data represent the mean ± SD of three independent experiments, with * *p* < 0.05 indicating statistical significance compared to the control (untreated cells). Compounds are designated as **1**, **3**, and **5** on the chart. The dashed line on the Y axis indicates 100% as control cells. (**b**). Analysis of changes in the mitochondrial membrane potential (MMP) of HCT 116, LoVo colon cancer cells treated with the investigated flavanone/chromanone derivatives (IC_50_ concentrations) using the fluorescent probe JC-1. The orange-red fluorescence of JC-1 aggregates is characteristic of non-apoptotic cells, whereas the green fluorescence of JC-1 monomers occurs in cells affected by PCD. The microscopic images were captured at a magnification of 20× with a Nikon Eclipse Te200 microscope with a ZEISS Axiocam 208 color microscope camera using a Nikon LWD Ph1 DL 20 × 0.40 lens.

Changes in mitochondrial potential in cancer cells exposed to the tested derivatives were monitored using the fluorescent probe JC-1. In non-apoptotic cells, JC-1 aggregates emit red-orange fluorescence. During changes in mitochondrial membrane potential, such as those associated with the initiation of PCD stages (both hyperpolarization and depolarization of mitochondrial membranes), JC-1 is converted to monomers, emitting green fluorescence. The results are presented in Figure 1a. Analysis of mitochondrial membrane potential (ΔΨm) in LoVo, HCT 116, SW620, HT-29, and Caco-2 cancer cells exposed to the tested derivatives revealed significant hyperpolarization or depolarization. Notably, most derivatives induced hyperpolarization, suggesting the initiation of early-stage PCD. Depolarization was observed only with the most potent derivative **1** in LoVo and Caco-2 cells, resulting in a ~20% and ~30% decrease in ΔΨm, respectively. The SW620 cell line exhibited the most pronounced sensitivity, with compounds **1** and **3** inducing mitochondrial membrane potential increases of approximately 40% and 60%, respectively. LoVo, HCT 116, and HT-29 cells showed slightly smaller but statistically significant hyperpolarization following 24 h incubation with the derivatives, reaching levels of approximately 20–30%. In the case of HCT 116 and SW620 lines, no statistically significant changes were observed after incubation with derivatives **1** and **5**, respectively.

The observed hyperpolarization or depolarization of mitochondrial membrane potential in cancer cells exposed to the tested derivatives suggests their pro-apoptotic potential and activation of PCD pathways. The intense hyperpolarization, typically associated with the early stages of PCD, may be attributed to oxidative stress and DNA damage induced by the derivatives. As noted by Zorov et al. [17], the hyperpolarization phase may coincide with increased ROS production. Numerous scientific reports have demonstrated the ability of flavanone/chromanone derivatives to target cancer cell mitochondria, leading to increased Bax/Bcl-2 ratios, the release of pro-apoptotic factors, and ultimately, ROS-mediated apoptosis [18,19]. Our results, obtained using the JC-1 probe to assess mitochondrial membrane potential, align with these findings. The observed changes in mitochondrial potential following incubation with the tested derivatives strongly suggest the activation of cell death pathways, possibly with the involvement of ROS.

The substantive supplementation of fluorometric measurements consists of images from fluorescence microscope imaging using the JC-1 probe. Figure 1b presents illustrative images obtained from HCT 116 and LoVo cell lines showing these ΔΨm changes in colorectal cancer cells.

### 2.3. Pro-Oxidant Properties of 3-Benzylidenechromanone/3-Benzylideneflavanone Derivatives: Changes in ROS/RNS Concentration

Traditional chemotherapy often induces ROS in cancer cells, leading to DNA, protein, and cellular damage. Agents like cisplatin and doxorubicin directly stimulate ROS production, ultimately triggering cell death [20,21].

**Figure 2:**
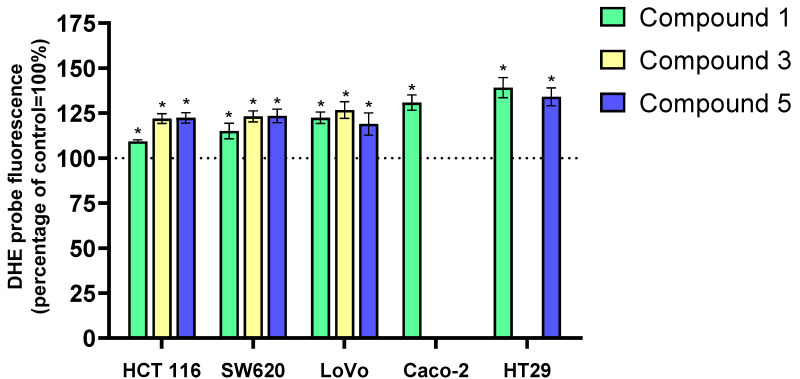
Analysis of changes in the levels of ROS (superoxide anion O_2_^•−^) of HCT 116, SW620, LoVo, Caco-2, and HT-29 colon cancer cells treated with the investigated flavanone/chromanone derivatives (IC_50_ concentrations) using the fluorescent probe dihydroethidium (DHE). The results are expressed as a percentage of the control, calculated by comparing the fluorescence intensity ratio of the test sample to the control (100%). Data represent the mean ± SD of three independent experiments, with * *p* < 0.05 indicating statistical significance compared to the control (untreated cells). Compounds are designated as **1**, **3**, and **5** on the chart. The dashed line on the Y axis indicates 100% as control cells.

Leveraging ROS in cancer therapy, coupled with inhibiting cancer cell antioxidant mechanisms, is a promising strategy. However, selective targeting of cancer cells remains a challenge. Ongoing research aims to optimize ROS-based therapies for improved efficacy and reduced toxicity [22,23]. The H_2_DCFD-DA and DHET probes were used to measure ROS levels in LoVo, HCT 116, SW620, HT-29, and Caco-2 cancer cells after 24 h of incubation with three selected compounds (**1**, **3**, **5**). The tested compounds primarily induced the generation of the superoxide anion radical (O_2_^─•^) in cancer cells, with a much smaller increase in hydrogen peroxide (H_2_O_2_). Exposure of cancer cells to *3*-benzylideneflavanone and *3*-benzylidenechromanone derivatives for 24 h increased O_2_^─•^ levels by approximately 25–40% compared to control cells. These changes were observed for all flavanone/chromanone derivatives and all cell lines. Only with derivative **1** and the HCT 116 and SW620 cell lines was a slightly smaller increase in O_2_^─•^ levels (10–15%) observed. The HT-29 and Caco-2 cell lines were more sensitive to ROS changes. In HT-29 cells, derivatives 1 and 5 increased O_2_^─•^ levels by approximately 40% and 35%, respectively. Similarly, Caco-2 cells exposed to derivative 1 showed a 30% increase in O_2_^─•^ levels compared to control cells (Figure 2).

**Figure 3:**
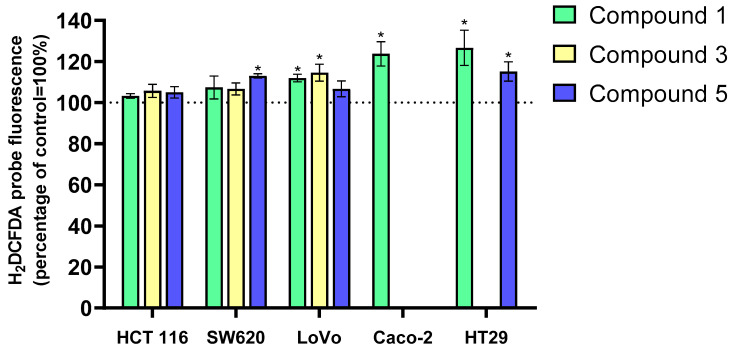
Analysis of changes in the levels of ROS (hydrogen peroxide H_2_O_2_) of HCT 116, SW620, LoVo, Caco-2, and HT-29 colon cancer cells treated with the investigated flavanone/chromanone derivatives (IC_50_ concentrations) using the fluorescent probe dihydroethidium (H_2_DCFDA). The results are expressed as a percentage of the control, calculated by comparing the fluorescence intensity ratio of the test sample to the control (100%). Data represent the mean ± SD of three independent experiments, with * *p* < 0.05 indicating statistical significance compared to the control (untreated cells). Compounds are designated as **1**, **3**, and **5** on the chart. The dashed line on the Y axis indicates 100% as control cells.

The kinetics of H_2_O_2_ (Figure 3) changes differed significantly from those of O_2_^─•^ in all cell lines. Increases in hydrogen peroxide levels were much smaller and observed only in certain experimental variants. Derivative 1 induced the largest increase in H_2_O_2_ levels, particularly in HT-29 and Caco-2 cells (approximately 25%). Interestingly, derivative 5 did not induce such a large increase in H_2_O_2_ levels in HT-29 cells, with changes around 15% compared to the control. Similarly, SW620 cells exposed to derivative 5 showed a 15% increase in H_2_O_2_ levels, but this derivative did not affect H_2_O_2_ levels in the other cancer cell lines. The LoVo cell line was sensitive to derivatives 1 and 3, showing a 10% increase in H_2_O_2_ levels after 24 h of incubation. In contrast, none of the tested flavanone/chromanone derivatives affected H_2_O_2_ levels in the HCT 116 cell line.

**Figure 4:**
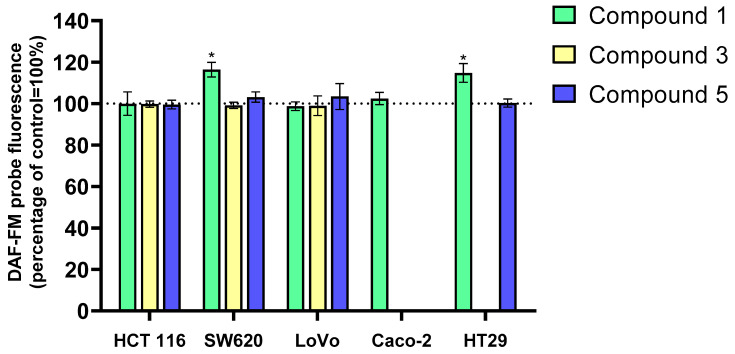
Analysis of changes in the levels of RNS of HCT 116, SW620, LoVo, Caco-2, and HT-29 colon cancer cells treated with the investigated flavanone/chromanone derivatives (IC_50_ concentrations) using the fluorescent probe 4-amino-5-methylamino-2′,7′-difluorofluorescein diacetate (DAF-FM). The results are expressed as a percentage of the control, calculated by comparing the fluorescence intensity ratio of the test sample to the control (100%). Data represent the mean ± SD of three independent experiments, with * *p* < 0.05 indicating statistical significance compared to the control (untreated cells). Compounds are designated as **1**, **3**, and **5** on the chart. The dashed line on the Y axis indicates 100% as control cells.

The effects of flavanone/chromanone derivatives **1**, **3**, and **5** on reactive nitrogen species (RNS) levels in colon cancer cells were also evaluated using the DAF-FM probe. The results are presented in Figure 4. Derivative **1** significantly increased RNS levels in HT-29 and SW620 cells, while no significant changes were observed with derivatives **3** and **5** in any of the tested cell lines. These results suggest that RNS contribute minimally to the overall antiproliferative and cytotoxic activity of the analyzed compounds.

A common chemotherapy strategy involves the use of pro-oxidant compounds that exert their anticancer effects by generating ROS in cancer cells and inducing oxidative stress. Many commonly used chemotherapy drugs, such as cisplatin and doxorubicin, generate ROS in cancer cells. Additionally, various metal complexes, phytochemicals, and small molecules are currently being investigated in preclinical and clinical studies for their potential anticancer activity through ROS induction [23,24,25].

While polyphenolic compounds are known for their antioxidant properties, as reported by Khan et al. [26], they can also exhibit pro-oxidant activity and induce oxidative stress in cancer cells, leading to their death. Quercetin serves as an excellent example of the dual nature of flavonoid activity. Depending on the concentration and cellular context, quercetin can exhibit both antioxidant and pro-oxidant properties. At higher concentrations, quercetin can induce ROS, thereby activating apoptotic pathways in cancer cells [27].

Our findings support the hypothesis that the anticancer effects of the studied flavanone/chromanone derivatives are mediated by oxidative stress induction in cancer cells. Previous studies have shown that structural modifications, such as the introduction of electron-withdrawing or electron-donating groups (e.g., methoxy groups), can enhance the pro-oxidant properties of chromanone and flavanone derivatives. These findings suggest that the design of flavanone/chromanone derivatives can be strategically optimized to maximize their pro-oxidant effects on cancer cells [28,29]. The structural characterization of the tested derivatives reveals a significant presence of pi double bonds, likely contributing to their pro-oxidant properties. Their susceptibility to fragmentation makes them ideal substrates for free radical reactions. Additionally, these π non-covalent double bonds can act as catalysts and attachment sites for free radicals, leading to a cascade of radical production.

One mechanism of ROS generation involves the autoxidation of flavanone derivatives. Molecular oxygen acts as a substrate in these reactions, undergoing one- or two-electron transfer reactions to produce superoxide anion radicals. Subsequently, certain portions of the flavonoid molecules, such as hydrogen atoms, can react with these superoxide radicals. This process involves the donation of a hydrogen atom from the flavonoid, resulting in the formation of hydrogen peroxide (H_2_O_2_) [30]. Studies have demonstrated that the carbonyl group at position 4 of the C ring influences the pro-oxidant activity of flavonoids [30]. Malarz et al. [31] demonstrated that thiosemicarbazones, structurally related to chromanones, generate ROS through redox-active complexes that induce cytotoxicity in cancer cells. These findings suggest that similar mechanisms may underlie the pro-oxidant activity of chromanone derivatives, particularly those capable of chelating metal ions.

Depending on the specific flavanone derivative, a highly reactive hydroxyl radical can be generated in the subsequent step of ROS production. Hydrogen peroxide, in the presence of Fe^2+^ iron ions, undergoes the Fenton reaction, leading to the formation of a hydroxyl radical. As one of the most potent free radical species, the hydroxyl radical significantly accelerates DNA damage and the formation of 8-oxo-2′-deoxyguanosine (8-oxo-dG), ultimately resulting in DNA mutations and cell death [24,26].

### 2.4. The Contribution of ROS to the Cytotoxicity of the Tested Pyrazoline Derivatives: MTT Test with Antioxidants

**Figure 5:**
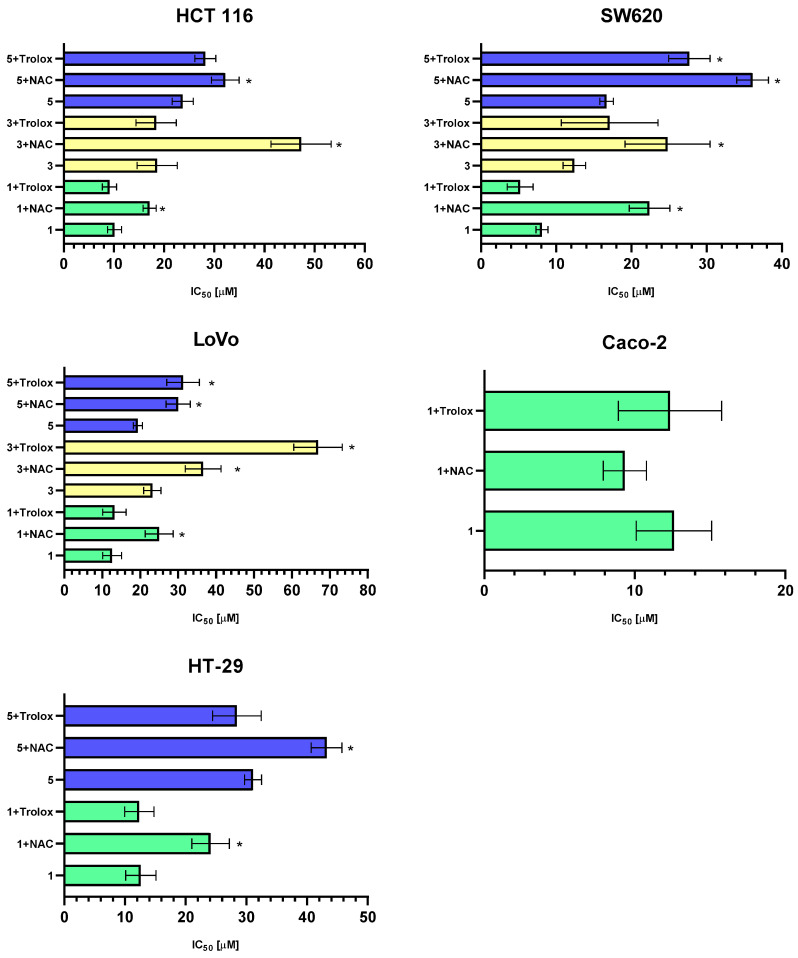
Changes in cytotoxic activity of the flavanone/chromanone derivatives **1**, **3,** and **5** toward cancer cells after 1 h of pre-incubation with NAC or Trolox or without antioxidants. The cells were incubated with the investigated compounds for 24 h. Results are expressed as the mean ± SD of three repeated experiments. IC_50_ values (µM) indicate the concentration of a compound required to reduce the fraction of surviving cells to 50% compared to the control probe (i.e., an untreated cell) with * *p* < 0.05 indicating statistical significance compared to cells without cells without pre-incubation with antioxidants. Compounds are designated as **1**, **3**, and **5** on the chart.

The results of our analysis on the pro-oxidant properties of the studied flavonoid derivatives clearly demonstrate their significant potential to induce oxidative stress in cancer cells. Moreover, the elevation of ROS levels was accompanied by a decrease in intracellular GSH. To investigate the role of ROS in the cytotoxic activity of flavonoid derivatives against cancer cells, LoVo cancer cells were pre-treated with antioxidants, NAC or a water-soluble derivative of vitamin E (Trolox), for 1 h before exposure to the studied compounds. Subsequently, the MTT assay was performed. Cytotoxicity results following 1 h of pre-incubation of cells with the antioxidants NAC and Trolox are presented in Figure 5, and the calculated IC_50_ concentrations for individual tumor lines are provided in Table 2.

The MTT assay results for the antioxidant-treated variants corroborate the data obtained from the ROS generation experiment in intestinal cancer cells, confirming the crucial role of free radicals in determining the total antiproliferative potential of the tested derivatives. In most experimental variants, pre-incubation with antioxidants significantly reduced the cytotoxic properties of the investigated complexes. NAC was more effective in neutralizing the pro-oxidant effects of the tested derivatives. Pre-incubation with NAC for 1 h significantly increased the IC50 value by approximately ~10–20 µM, indicating a reduction in the overall cytotoxic and antiproliferative activity of the analyzed derivatives. A synthetic derivative of vitamin E (Trolox) exhibited a significantly weaker inhibition of ROS-dependent cytotoxic properties. Statistically significant increases in the IC_50_ value were observed for compound **5** in the SW620 cell line and for compounds **3** and **5** in the LoVo cell line.

The differences in the effectiveness of NAC and Trolox in inhibiting the antiproliferative and cytotoxic properties of the tested derivatives against the cancer cells used may be attributed to their specific mechanisms of action and sites of activity. The antioxidant properties of NAC are primarily due to its ability to increase intracellular GSH levels, thereby reducing ROS levels. Additionally, NAC can directly interact with and inactivate free radicals, such as the hydroxyl radical, hydrogen peroxide, and hypochlorous acid. This free radical scavenger primarily acts in the cytoplasm of cells [32,33]. It is possible that the observed reduction in the antiproliferative properties of the tested derivatives resulted from the partial replenishment of the GSH pool in cancer cells, leading to more effective scavenging of generated ROS. This hypothesis is supported by our findings using the MCB probe, which demonstrated that the analyzed flavonoid conjugates caused a decrease in GSH concentration in cancer cells.

Vitamin E, on the other hand, is a strong antioxidant that works primarily in cell membranes. As a fat-soluble molecule, it easily penetrates the lipid bilayer of the cell membrane, protecting the particularly sensitive unsaturated fatty acids from the harmful effects of ROS [34,35]. The MTT cytotoxicity results after Trolox pre-incubation suggest that the tested compounds enter cells and exert their pro-oxidant effects in the cytosol, where ROS are generated. Free radical reactions primarily target intracellular components, such as DNA, rather than directly targeting cell membranes and causing lipid peroxidation [17,25].

### 2.5. Pro-Oxidant Properties of 3-Benzylideneflavanone/3-Benzylidenechromanone Analogs: Changes in the Concentration of Reduced Glutathione GSH

As a key component of the cellular antioxidant system, GSH plays a multi-faceted role in regulating cell growth, proliferation, adaptation, and death. Dysregulation of GSH levels has been associated with the pathogenesis of many human diseases, including cancer [22]. Numerous studies have reported higher glutathione levels in cancer cells compared to normal cells. Elevated GSH levels in cancer cells have been associated with increased proliferation, tumor metastasis, and multi-drug resistance [36,37]. GSH, a potent antioxidant, exhibits a dual role in cancer biology. On the one hand, it acts as a protective mechanism against oxidative stress by scavenging free radicals. Conversely, its elevated levels in various cancer cells (e.g., melanoma, hepatocancer, breast, colon, pancreatic, and lung cancer) have been implicated in increased cancer progression, decreased chemotherapy efficacy, and poor patient prognosis [38,39].

Given these properties, GSH has emerged as a potential target for anticancer therapy. A primary strategy involves reducing GSH levels in cancer cells to enhance their sensitivity to treatment [40]. A pivotal aspect of GSH’s role in cancer is its involvement in chemotherapy, which often induces oxidative stress in cancer cells through pro-oxidant chemotherapeutic drugs (including anthracyclines, alkylating agents, and platinum coordination complexes) or ionizing radiation. Elevated levels of GSH and related enzymes in cancers enable them to more effectively detoxify anticancer drugs and neutralize reactive oxygen species (ROS) [41].

The monochlorobimane (MCB) probe was used to assess the effects of the tested compounds on intracellular GSH levels in cancer cells. 

**Figure 6:**
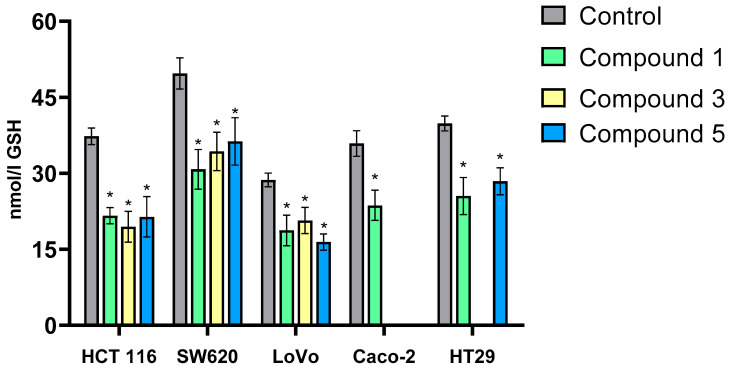
Analysis of changes in the levels of GSH of HCT 116, SW620, LoVo, Caco-2, and HT-29 colon cancer cells treated with the investigated flavanone/chromanone derivatives (IC_50_ concentrations) using the fluorescent Monochlorobimane (MCB) probe. The results are expressed as a percentage of the control, calculated by comparing the fluorescence intensity ratio of the test sample to the control (100%). Data represent the mean ± SD of three independent experiments, with * *p* < 0.05 indicating statistical significance compared to the control (untreated cells). Compounds are designated as **1**, **3**, and **5** on the chart.

Exposure of colon cancer cells to the tested derivatives for 24 h significantly reduced intracellular GSH levels compared to control cells in all experimental variants (Figure 6). Comparative analysis of the results for individual cell lines revealed that the three flavanone/chromanone derivatives exhibited similar effectiveness in reducing GSH levels. The percentage decrease in GSH levels was comparable for derivatives **1**, **3**, and **5** in each cell line. The HT-29 and Caco-2 cell lines, which demonstrated lower sensitivity to the derivatives, showed a 25–30% decrease in GSH levels after incubation with derivatives **1** and **5** (HT-29) or derivative **1** (Caco-2). In contrast, the SW620 and HCT 116 cell lines exhibited a more pronounced decrease in GSH levels. Exposure of SW620 cells to compound **1** resulted in a 40% decrease in GSH levels, while compounds **3** and **5** induced a slightly smaller (30%) decrease. Similar results were observed in the HCT 116 cell line. LoVo cells also showed a significant (30–35%) decrease in GSH levels after incubation with compounds **1**, **3**, and **5.**

Our findings regarding changes in GSH levels in colon cancer cells align with existing literature. Monteleone et al. [42] demonstrated that inhibiting GSH synthesis sensitized neuroblastoma stem cells to etoposide and improved chemotherapy effectiveness. Additionally, GSH participates in regulating cancer cell death through necroptosis [43] and ferroptosis [44]. Studies have shown that reducing GSH levels or its synthesizing enzymes can enhance cell death [45,46,47].

Our results suggest that reducing GSH levels in cancer cells, a strategy facilitated by the flavanone/chromanone derivatives we investigated, contributes to their anticancer properties. This effect is further amplified by the strong pro-oxidant properties of these derivatives.

Based on the obtained results regarding the induction of oxidative stress in cancer cells by the tested derivatives, it appears that their pro-oxidant effects arise not only from direct ROS generation but also from indirect inhibition of the key antioxidant GSH. The significant attenuation of the cytotoxic and antiproliferative properties of derivatives **1**, **3**, and **5** upon pre-incubation with NAC further corroborates the involvement of oxidative stress in their anticancer activity.

### 2.6. Induction of Autophagy in Cancer Cells by 3-Benzylideneflavanone/3-Benzylidenechromanone Analogs

**Figure 7:**
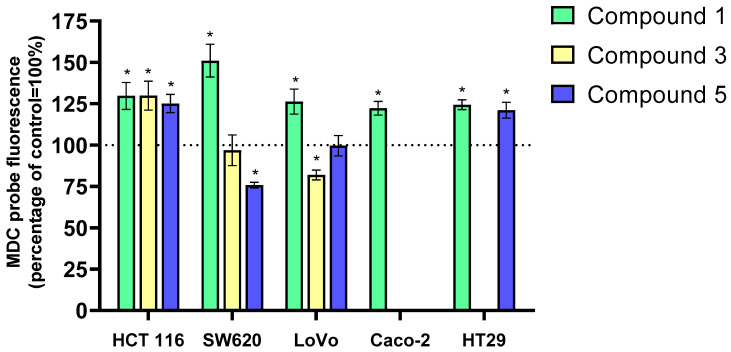
Analysis of induction of autophagy in HCT 116, SW620, LoVo, Caco-2, and HT-29 colon cancer cells treated with the investigated flavanone/chromanone derivatives (IC_50_ concentrations) using the fluorescent probe Monodansylcadaverine (MDC). The analysis was performed based on the level of MDC accumulation in autophagolysosomes. The results are expressed as a percentage of the control, calculated by comparing the fluorescence intensity ratio of the test sample to the control (100%). Data represent the mean ± SD of three independent experiments, with * *p* < 0.05 indicating statistical significance compared to the control (untreated cells). Compounds are designated as **1**, **3**, and **5** on the chart. The dashed line on the Y axis indicates 100% as control cells.

Autophagy is a conserved cellular process essential for maintaining cellular homeostasis. It eliminates damaged organelles and proteins and can both suppress and promote tumorigenesis depending on the context [48,49]. Autophagy was assessed using the fluorescent probe monodansylcadaverine (MDC), which accumulates in autophagolysosomes. The results of the analysis of autophagy induction in cancer cells based on changes in MDC content in autophagolysosomes are presented in Figure 7.

Incubation of LoVo, HCT 116, SW620, HT-29, and Caco-2 cancer cells with the tested flavanone/chromanone derivatives 1, 3, and 5 predominantly induced autophagy. The most pronounced increase in MDC intensity, indicative of autophagic cell death, was observed in the SW620 cell line treated with derivative **1**, amounting to approximately 40% compared to the control. Interestingly, the other two derivatives did not induce autophagy in this cell line. For compound **3**, no significant changes in MDC fluorescence intensity were detected, while compound **5** exhibited a 20% decrease compared to the control, suggesting the onset of apoptosis.

A similar trend of autophagy/apoptosis induction was observed in the LoVo cell line. Derivative **1** again led to increased MDC fluorescence, albeit to a lesser extent (approximately 20%) than in SW620 cells. Treatment with derivative **3** induced programmed cell death in LoVo cells, as evidenced by a 20% decrease in MDC fluorescence. No significant changes were observed with compound **5**.

The HCT 116 cell line exhibited a marked enhancement of autophagy upon exposure to all three derivatives. A clear, statistically significant increase in MDC fluorescence intensity of approximately 25–30% was observed compared to the control.

In the HT-29 cell line, derivatives **1** and **5** increased MDC fluorescence by 25% and 20%, respectively, compared to the control. Similar results were obtained for derivative **1** in the Caco-2 cell line, with a 25% increase in MDC fluorescence.

Based on these findings, derivative **1** appears to be the most potent autophagy inducer among the tested compounds. It increased MDC fluorescence intensity in all five colon cancer cell lines (LoVo, HCT 116, SW620, HT-29, and Caco-2), indicating accumulation in autophagolysosomes. The induction of autophagy or apoptosis by derivatives **3** and **5** was cell-line-dependent.

When discussing the antiproliferative properties of flavanone derivatives and the types of cell death they induce, it is crucial to note their ability to simultaneously activate apoptosis and autophagy. The synergistic effect of these processes enhances their anticancer potential [50].

Recent research by Chang et al. [50] suggests that the induction of autophagy in cancer cells by flavanone derivatives may be linked to their ability to generate ROS, which subsequently activate autophagic pathways. Based on these findings, we hypothesize that the oxidative stress induced by our studied derivatives may contribute to the activation of autophagic and apoptotic responses, ultimately leading to cancer cell death.

A key aspect to consider regarding autophagy induction by our derivatives is their impact on the PI3K/Akt/mTOR signaling pathway. This pathway plays a pivotal role in regulating cell growth and survival, and its inhibition can trigger autophagy. mTOR, a critical regulator of autophagy, can initiate autophagy when inhibited, leading to cancer cell death [51].

Given that previous studies have demonstrated the ability of certain flavone derivatives to effectively inhibit the mTOR pathway, inducing autophagy and subsequent cancer cell death, further investigation into the effects of our derivatives on this pathway is warranted [52,53,54].

### 2.7. Evaluation of Genotoxic Properties of 3-Benzylideneflavanone/3-Benzylidenechromanone Analogs Using the Comet Assay

Genotoxic chemotherapeutic agents target tumor cells by forming DNA adducts, inhibiting replication, and indirectly inducing DNA damage through DNA repair enzyme inhibition or ROS generation [55].

The level of DNA damage in the HCT 116, SW620, LoVo, Caco-2, and HT-29 cell lines was evaluated using a standard comet assay (single-cell electrophoresis in agarose gel). 

**Figure 8:**
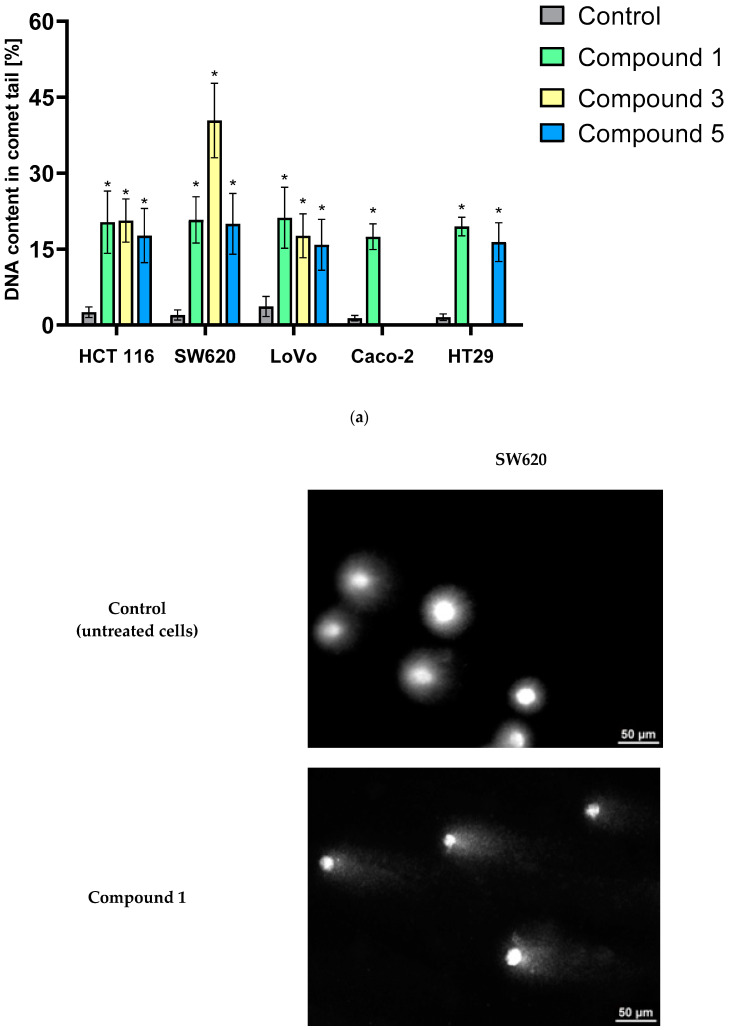

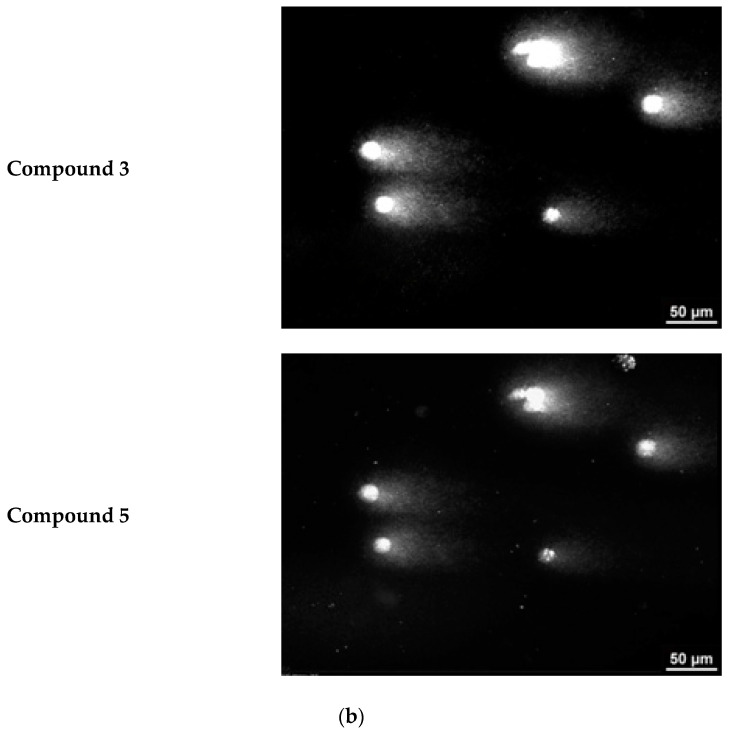
(**a**). Analysis of DNA damage levels in HCT 116, SW620, LoVo, Caco-2, and HT-29 colon cancer cells treated with the investigated flavanone/chromanone derivatives (IC_50_ concentrations) using alkaline comet method (single-cell electrophoresis). Data represent the mean ± SD of three independent experiments, with * *p* < 0.05 indicating statistical significance compared to the control (untreated cells). Each time, 100 cells were analyzed from a slide. Compounds are designated as **1**, **3**, and **5** on the chart. (**b**). Analysis of DNA damage levels of SW620 colon cancer cells treated with the investigated flavanone/chromanone derivatives (IC_50_ concentrations) using alkaline comet method (single-cell electrophoresis). The microscopic images were captured at a magnification of 20× with a Nikon Eclipse fluorescence microscope equipped with a 4910 COHU video camera.

Comet assays revealed that all analyzed derivatives (**1**, **3**, and **5**) exhibited potent genotoxic properties (Figure 8a). After incubating HCT 116, SW620, LoVo, Caco-2, and HT-29 cancer cell lines with these compounds for 12 h, a statistically significant increase in the percentage of cells with damaged DNA was observed in all experimental groups. The most pronounced DNA damage was detected in the SW620 cell line exposed to compound **3**, with an approximately 40% increase in DNA content in the comet tail compared to the control group. The remaining derivatives also demonstrated a clear ability to induce DNA damage, causing an increase of approximately 15–20% compared to the control cells. These findings unequivocally indicate the high genotoxicity of the tested compounds.

Representative images from the comet assay are shown in Figure 8b. The comet “tail” is clearly visible in cells with damaged DNA. The length of the comet “tail” is directly proportional to the percentage of damaged cell DNA.

The obtained results suggest that the DNA-damaging potential of the analyzed flavone/chromanone derivatives in cancer cells is one of the mechanisms underlying their anticancer activity. According to the available scientific literature, the genotoxic activity of these derivatives can be realized at several molecular stages, including direct interaction of flavonoid/chromanone derivatives with DNA [56] or indirectly, even through the inhibition of enzymes involved in DNA repair [57]. In our previous work [58], we demonstrated that two compounds, 5′-(3-methylphenyl)-2-phenyl-4′,5′-dihydro-4*H*-spiro[chromano-3,3′-pirazol]-4-one and 5′-(4-methylphenyl)-2-phenyl-4′,5′-dihydro-4*H*-spiro[chromano-3,3′-pirazol]-4-one molecules, structurally similar to the studied derivatives, effectively interacted with poly-ADP ribose polymerase (PARP), leading to its inactivation and degradation. These results are consistent with recent findings by Hassan et al. [57], who showed that certain stilbenoid–flavanone hybrids are capable of, among other effects, dose-dependent cleavage of PARP.

In light of our studies and the genotoxic properties of the investigated derivatives, strong pro-oxidant activity also appears to be crucial. As demonstrated by our studies using antioxidants (NAC and Trolox), the generation of ROS in cancer cells is a key determinant of the anticancer potential of our flavone/chromanone derivatives. Therefore, one of the primary mechanisms of action of these compounds (**1**, **3,** and **5)** is undoubtedly their ability to generate ROS. It can therefore be assumed that the potential of flavonoid derivatives to induce the formation of a highly reactive molecule, such as a hydroxyl radical, may accelerate DNA damage through the production of 8-oxo-2′-deoxyguanosine (8-oxo-dG). The presence of such modified nitrogen bases leads to DNA mutations, and ultimately, cell death [24,26].

### 2.8. Analysis of Cell Cycle Phase Changes in Cancer Cells Exposed to 3-Benzylideneflavanone/3-Benzylidenechromanone Analogs 

The cytostatic activity of anticancer compounds is primarily determined by their effect on the cell cycle. Many available chemotherapeutic agents, due to their genotoxic properties, damage the DNA of cancer cells, consequently causing a cell cycle arrest in the G2/M phase. 

**Figure 9:**
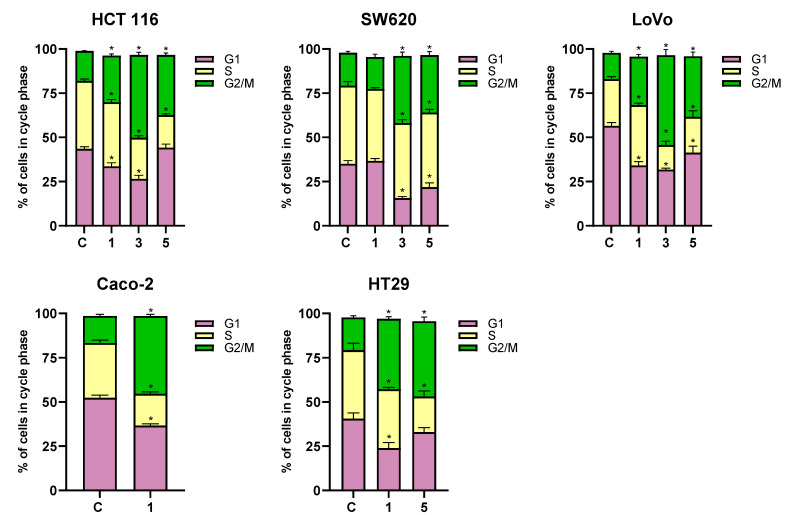
Cell cycle distribution of HCT 116, SW620, LoVo, Caco-2, and HT-29 colon cancer cells treated for 24 h with the investigated flavanone/chromanone derivatives (**1**, **3**, **5**; IC_50_ concentrations) and control (C) untreated cells. The percentage of DNA in a particular phase/point of the cell cycle was calculated from the DNA histograms using available software (FlowJo 10.10). DNA was stained using propidium iodide/RNAseA solution. Representative plot from each time point is shown (n = 3). Data represent the mean ± SD of three independent experiments, with * *p* < 0.05 indicating statistical significance compared to the control (untreated cells).

**Figure 10:**
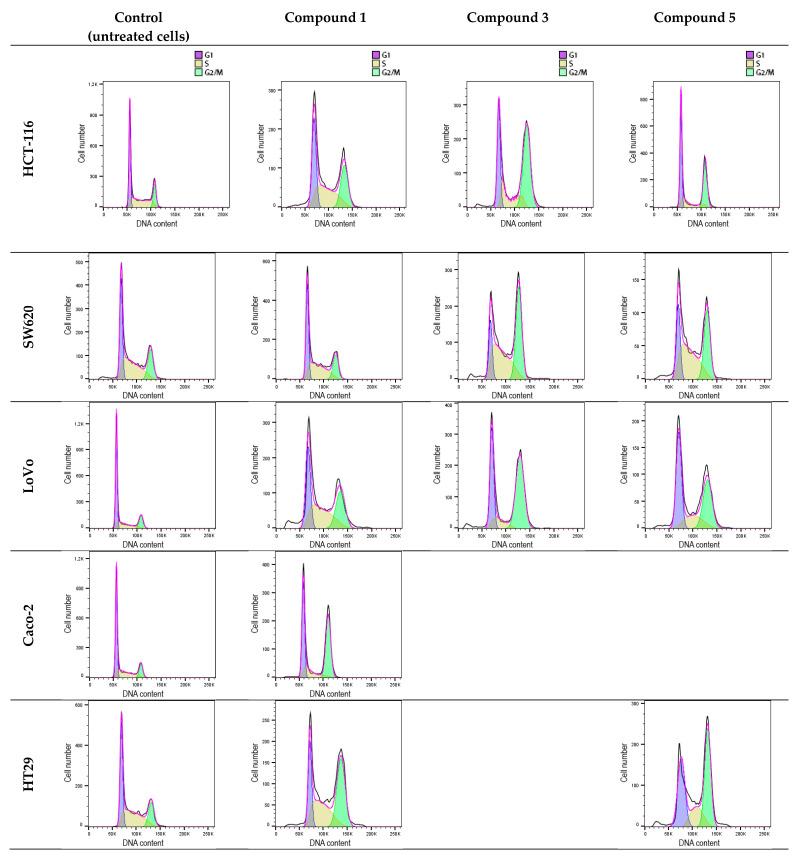
Flow cytometry plots from HCT 116, SW620, LoVo, Caco-2, and HT-29 colon cancer cells treated for 24 h with the investigated flavanone/chromanone derivatives (IC_50_ concentrations). DNA was stained using propidium iodide/RNAse solution. Representative plot from each time point is shown (n = 3).

DNA histograms derived from the cytometric analysis of HCT 116, SW620, LoVo, Caco-2, and HT-29 cells exposed to flavanone/chromanone derivatives revealed significant cell cycle disturbances (Figure 9 and Figure 10). All tested derivatives induced G2/M cell cycle arrest, which may underlie their cytostatic effect. This effect is likely attributable to the compounds’ pro-oxidant and genotoxic properties. The extent of alterations in the individual G1, S, and G2/M cell cycle fractions of cancer cells varied across different cell lines.

In the case of the HCT 116 line, the most significant changes were observed for compound **3**, which induced a substantial increase in the G2/M phase cell fraction by approximately 30% compared to the control. Derivative **5** exhibited slightly weaker cytostatic activity, resulting in a 20% increase in the G2/M cell fraction compared to the control. Interestingly, derivative **1**, characterized by the strongest cytotoxic properties, induced the weakest G2/M phase cell cycle arrest in HCT 116 cells, at approximately 10%.

A similar trend in the cytostatic activity of the analyzed flavanone/chromanone derivatives was observed for the LoVo line. Again, the greatest increase in the G2/M phase cell percentage was observed for derivative **3**, with an approximately 35% increase compared to control cells. Derivative **5** induced a cell cycle arrest in LoVo cells at a level of approximately 20% compared to the control, while derivative 1 exhibited the weakest cytostatic activity, with an approximately 10% increase in the G2/M phase cell percentage.

The SW-620 line displayed slightly lower sensitivity to cell cycle arrest. Notably, for the most cytotoxic derivative 1, no statistically significant changes in cell cycle parameters or G2/M phase arrest were observed. Importantly, this is the only experimental condition where no statistically significant inhibition of the cancer cell division cycle was detected. For derivatives **3** and **5**, increases in the G2/M cell fraction were observed by approximately 20% and 13%, respectively, compared to the control.

A clear G2/M phase cell cycle arrest was observed for the HT-29 line exposed to derivatives **1** and **5**. Both compounds exhibited comparable cytostatic effects. A 24 h incubation with derivatives **1** and **5** resulted in a 25% increase in the G2/M phase cell percentage for both compounds.

Derivative **1** maintained its potent cytostatic activity against the Caco-2 line. Incubation of cells with this compound led to a clear G2/M phase cell cycle arrest, manifested by a 30% increase in cells in this phase compared to control cells.

As indicated by previous studies, the cytostatic properties of flavanone/chromanone derivatives and their ability to induce G2/M phase cell cycle arrest in cancer cells are complex processes, likely involving multiple molecular pathways [14,59,60]. In our studies, the pro-oxidant properties of the analyzed flavanone/chromanone derivatives appear to be a crucial factor in cell cycle inhibition. Oxidative stress can damage cellular components, including DNA, thereby activating cell cycle checkpoints and promoting apoptosis. This hypothesis is supported by the findings of Su et al. [61], who demonstrated that fisetin increases ROS levels, leading to apoptosis and cell cycle arrest in oral cancer cells.

Importantly, our previous studies [14] using 3-benzylidenechromanones indicated that these compounds can directly intercalate into DNA, causing DNA damage and, consequently, G2/M phase cell cycle arrest and apoptosis.

When considering the mechanisms of cytostatic activity of the studied flavanone/chromanone derivatives, it is essential to consider their potential effects on key cell cycle regulators. Recent studies by Gao et al. [62] have shown that flavonoids induce G2/M arrest by activating checkpoint kinase 2 (Chk2), a key cell cycle regulator. Additionally, studies by Li et al. [63] provide valuable insights into the effects of flavanone/chromanone derivatives on the activity/expression of key regulatory proteins involved in the cell cycle. As the authors emphasize, the PI3K-Akt-mTOR pathway and, consequently, the activity/expression of cyclin B1, a key protein for the G2/M phase, may be potential targets for flavonoid derivatives.

## 3. Materials and Methods

### 3.1. Cell Culture, Cell Passaging, and Biological Material

The evaluation of the antitumor properties of the tested chroman-4-one derivatives was performed on 5 human adherent colon cancer lines: HCT 116 (ATCC^®^ CCL-247™, colorectal cancer, Dukes’ type A), SW620 (ATCC^®^ CCL-227™, colorectal adenocancer, Dukes’ type C), LoVo (ATCC^®^ CCL-229™, colorectal adenocancer, Dukes’ type C, grade IV), Caco-2 (ATCC^®^ HTB-37™, colorectal adenocancer, Dukes’ type B), and HT-29 (ATCC^®^ HTB-38™, colorectal adenocancer, Dukes’ type C). All these cell lines were purchased from American Culture Collection (ATCC), Manassas, VA, USA. The selection of cell lines was dictated by genetic differences and the diversity of molecular subtypes of individual cell lines. Moreover, two adherent cell lines were used as a model of normal cells in studies on the cytotoxicity of the tested derivatives: human foreskin fibroblast Hs68 (ATCC^®^ CRL-1635™) and human microvascular endothelial cell culture HMEC-1 (ATCC^®^ CRL-3243™).

HMEC-1 cells were cultured in MCDB 131 (Corning Life Sciences, Corning, NY, USA) culture medium supplemented with 10% fetal bovine serum (Gibco, Grand Island, New York, NY, USA) and antibiotics (10 U/mL penicillin and 0.5 mg/mL streptomycin) (Gibco, Grand Island, New York, NY, USA). The culture medium was enhanced with L-glutamine (10 M), hydrocortisone (1 μg/mL), and epidermal growth factor EGF (10 ng/mL) (Millipore, Burlington, MA, USA).

HCT 116, SW620, and LoVo cancer cell lines were cultured in Dulbecco’s minimal essential medium (DMEM, Lonza, Visp, Switzerland) culture medium supplemented with 10% fetal bovine serum and antibiotics (10 U/mL penicillin and 0.5 mg/mL streptomycin). The Caco-2 cell line was cultured in Eagle’s minimum essential medium (EMEM, Sigma-Aldrich; St. Louis, MO, USA) culture medium supplemented with 20% fetal bovine serum and antibiotics (10 U/mL penicillin and 0.5 mg/mL streptomycin). HT-29 tumor cells were cultured in RPMI (Thermo Fisher Scientific, Waltham, MA, USA) culture medium supplemented with 10% fetal bovine serum and antibiotics (10 U/mL penicillin and 0.5 mg/mL streptomycin).

Cell culture was performed under sterile conditions, in sterile, disposable culture vessels. Cells were cultured under standard conditions in a CO_2_ incubator (temperature 37 °C, 95% air, 5% CO_2_, 100% relative humidity). Cells from all lines were maintained in the logarithmic growth phase via regular passages (2–3 times a week) to new culture vessels after the culture had reached approximately 80% confluence. The cell monolayer, after removing the culture medium, was washed with 0.9% NaCl. Then, 0.25% trypsin solution with EDTA (Gibco, Grand Island, New York, NY, USA) was added in an amount appropriate for the surface of a given culture vessel (300–500 μL). Cells were incubated with trypsin for 3–5 min in a CO_2_ incubator, and the trypsinization process was monitored under a microscope. After completion of trypsinization, the culture medium was added in a volume appropriate to the size of the new culture vessel, and the suspension, after thorough mixing, was transferred to new, sterile culture vessels. Cells from individual lines were seeded on culture plates/dishes at a density appropriate for a given experiment.

### 3.2. Determination of Cell Viability via Metabolic Microplate Spectrophotometric Assay with MTT

Cell viability was determined using the metabolic microplate spectrophotometric assay with Methylthiazolyl diphenyltetrazolium bromide (MTT) (Sigma-Aldrich; St. Louis, MO, USA). The MTT method belongs to metabolic tests for determining cell viability and consists of measuring the absorbance of a violet solution of formazan, a product of the reduction of a water-soluble yellow MTT by oxidoreductive enzymes (mainly mitochondrial succinate dehydrogenase) of living cells. The amount of the reaction product (water-insoluble formazan crystals) is proportional to the number of living and metabolically active cells in the sample. In the case of damaged or dead cells, formazan is formed in smaller amounts, or it is not formed. The violet solution obtained after dissolving formazan crystals in DMSO shows maximum absorbance at a wavelength of λ = 580 nm.

The appropriate number of cells for a given cell line (6–8 × 10^3^ cells/mL) were seeded onto colorless, sterile 96-well microplates (Nunc, Roskilde, Denmark) in the number appropriate for a given cell line. After 24 h of culture, various concentrations of the studied compounds (10^−7^–10^−5^ M) freshly prepared in DMSO (final concentration DMS < 0.1%) and diluted with complete culture medium were added and incubated in a CO_2_ incubator for 24 h. After this time, the medium was removed; the cell monolayer was washed twice with sterile PBS solution; and fresh culture medium was added to the microplate wells. After 48 h of cell culture, the MTT test was performed. A volume of 50 μL of tetrazolium salt solution (final concentration 0.05 mg/mL) was added to each microplate well. The microplate was placed for 3–4 h (depending on the cell line) in a CO_2_ incubator. After incubation, 100 μL of DMSO was added to the wells of the microplate to dissolve the purple formazan crystals formed in the living cell environment. The absorbance of the formazan solution, after gentle mixing, was read at a wavelength of λ = 580 nm and a reference wavelength of λ = 720 nm (Biotek Power Wave HT, Biotek Instruments, Winooski, VT, USA). The percentage of living cells was calculated by comparing the absorbance value of the tested samples with the absorbance value of the control (cells not treated with compounds). The absorbance of the control was taken as 100%. Based on the obtained results, cell survival curves were plotted using the GraphPad Prism computer program, and the IC_50_ concentration of compounds that reduced the percentage of living cells by 50% was calculated. Moreover, cisplatin (Sigma-Aldrich, St. Louis, MO, USA) was used as a reference compound, and identical incubation conditions were used as described above. 

### 3.3. Changes in the Mitochondrial Potential of Cancer Cells Using the Fluorescent Probe JC-1

Mitochondrial transmembrane potential (MMP, ΔΨm) is generated by the electrical potential across the inner mitochondrial membrane. The MMP measurement is useful for assessing mitochondrial function and detecting mitochondrial membrane dysfunction, such as depolarization or hyperpolarization. Decreased MMP may be associated with the generation of free radicals and induction of the inner (mitochondrial) apoptotic pathway [64].

5,5,6,6-Tetrachloro-1,1,3,3-tetraethylbenzimidazolylcarbocyanine iodide (JC-1) is one of the most reliable fluorescent probes used to assess changes in mitochondrial membrane potential (ΔΨ). In living cells with intact mitochondrial membranes, the lipophilic, cationic dye JC-1 penetrates the negatively charged mitochondrial matrix as a monomer, where it forms fluorescent aggregates with red-orange fluorescence (λ_ex_ = 514 nm, λ_em_ = 590). During depolarization of the mitochondrial membrane or in apoptotic cells, JC-1 remains in the form of monomers that exhibit green fluorescence (λ_ex_ = 514 nm, λ_em_ = 529 nm). The fluorescence ratio of aggregates (λ_em_ = 590 nm) and monomers (λ_em_ = 529 nm)—590 nm/529 nm—reflects the level of damage to the mitochondrial membrane. 

Cells were seeded in black 96-well fluorometric microplates at a density of 10 × 10^3^ cells/mL. After 24 h of cell culture, the cells were treated with the investigated compounds at their IC_50_ concentrations for another 12 h. Following treatment, the medium was removed, and the cell monolayers were washed twice with HBSS (140 mM NaCl, 5 mM KCl, 0.8 mM MgCl_2_, 1.8 mM CaCl_2_, 1 mM Na2HPO4, 10 mM HEPES, and 1% glucose). Then, 50 μL of a JC-1 solution in HBSS was added to each well, and the cells were incubated for 30 min in the dark at 37 °C (final JC-1 concentration 5 μM). After removing the fluorescent dye and replacing it with fresh HBSS, fluorescence measurements were taken using a Perkin-Elmer LS-5B spectrofluorometer (Perkin-Elmer LS-5B spectrofluorometer, Waltham, MA, USA). The excitation and emission wavelengths for JC-1 monomers were 514 nm and 529 nm, respectively, while for aggregates, they were 514 nm and 590 nm. The results were expressed as a percentage change in the fluorescence ratio of JC-1 aggregates to JC-1 monomers compared to the control, which was set at 100%.

As a positive control, cells treated with 5 μM CCCP (m-chlorophenylhydrazone) (Sigma-Aldrich; St. Louis, MO, USA) were used. CCCP is an organic ionophore that acts as a mitochondrial uncoupler. By transporting protons across the inner mitochondrial membrane, CCCP disrupts the proton gradient necessary for ATP synthesis via oxidative phosphorylation. As a result, the electrochemical potential of the inner mitochondrial membrane is reduced, simulating mitochondrial damage.

In addition, to visualize changes in the mitochondrial potential of cancer cells incubated with the flavanone derivatives tested, JC-1 fluorescence images were taken using a Nikon Eclipse Te200 microscope (Tokyo, Japan) with an Axiocam 208 color microscope camera (ZEISS, Oberkochen, Germany). Cells were seeded at a density of 3 × 10^5^ cells onto sterile 35 mm diameter dishes and cultured for 24 h to allow them to reach the logarithmic growth phase. Following this, the test compounds were added (IC_50_ concentration). After 12 h of incubation, the compounds were removed, and JC-1 probes were added to the cell environment under the experimental conditions described above. Images were taken with a Nikon LWD Ph1 DL 20 × 0.40 lens (Tokyo, Japan).

### 3.4. Reactive Nitrogen Species: Measurement of Changes in Nitric Oxide Levels in Cancer Cells Using the DAF-FM Probe

Diaminofluorescein-FM diacetate (DAF-FM diacetate, Invitrogen, Carlsbad, CA, USA), a cell-permeable fluorescent probe, is widely utilized for the detection and quantification of reactive nitrogen species (RNS), primarily nitric oxide (NO). In its native state, DAF-FM exhibits negligible fluorescence. Upon interaction with NO, however, it undergoes a chemical transformation resulting in the formation of a highly fluorescent benzotriazole derivative with excitation and emission maxima at 495 and 515 nm, respectively.

Cells were seeded in black 96-well fluorometric microplates at a density of 10 × 10^3^ cells/mL. After 24 h of cell culture, the cells were treated with the investigated compounds at their IC_50_ concentrations for another 24 h. Following compound incubation, the medium was removed, and the cell monolayers were washed twice with HBSS (140 mM NaCl, 5 mM KCl, 0.8 mM MgCl_2_, 1.8 mM CaCl_2_, 1 mM Na_2_HPO_4_, 10 mM HEPES, and 1% glucose). A volume of 50 µL of DAF-FM diacetate solution in HBSS (final concentration 5 μM) was added to each well, and the cells were incubated for 30 min in the dark at 37 °C. The probe was then replaced with fresh HBSS buffer, followed by an additional 30 min incubation to facilitate complete intracellular de-esterification of DAF-FM diacetate. Fluorescence measurements were conducted using a Perkin-Elmer LS-5B spectrofluorometer (MA, USA) at the following excitation and fluorescence emission wavelengths: λ_ex_ = 495 nm/λ_em_ = 515 nm. The fluorescence intensity of control cells was arbitrarily assigned a value of 100%. The results were expressed as percentages of control, calculated by comparing the fluorescence intensity ratio of the test samples to controls (100%). The fluorescence of samples was denoted as Δf in relation to the control.

### 3.5. Reactive Oxygen Species: Measurement of Changes in Superoxide Anion Levels in Cancer Cells Using the DHE Probe

Superoxide anion (O_2_^•−^) levels were quantified using a microplate spectrofluorometric assay with dihydroethidium (DHE) (Life Technologies, Carlsbad, CA, USA). DHE is a well-established fluorogenic probe for intracellular superoxide detection [65]. Upon oxidation to ethidium, DHE exhibits a fluorescence shift from blue to red, enabling its use as a sensitive indicator of reactive oxygen species (ROS) within cells. The reaction between superoxide and DHE produces a highly specific red fluorescent product, 2-hydroxyethidium (2-OH-E ^+^).

Cells were seeded in black 96-well fluorometric microplates at a density of 10 × 10^3^ cells/mL. After 24 h of cell culture, the cells were treated with the investigated compounds at their IC_50_ concentrations for another 24 h. Following compound incubation, the medium was removed, and the cell monolayers were washed twice with HBSS (140 mM NaCl, 5 mM KCl, 0.8 mM MgCl_2_, 1.8 mM CaCl_2_, 1 mM Na_2_HPO_4_, 10 mM HEPES, and 1% glucose). A volume of 50 µL of DHE solution in HBSS (final concentration 5 μM) was added to each well, and the cells were incubated for 30 min in the dark at 37 °C. After incubation with the DHE, fluorescence measurements were conducted using a Perkin-Elmer LS-5B spectrofluorometer (Waltham, MA, USA) at the following excitation and fluorescence emission wavelengths: λ_ex_ = 498 nm/λ_em_ = 582 nm. The fluorescence intensity of control cells was arbitrarily assigned a value of 100%. The results were expressed as percentages of control, calculated by comparing the fluorescence intensity ratio of the test samples to controls (100%). The fluorescence of samples was denoted as Δf in relation to the control.

### 3.6. Reactive Oxygen Species: Measurement of Changes in Hydrogen Peroxide Levels in Cancer Cells Using the H_2_DCFDA Probe

Cellular H_2_O_2_ levels were assessed using 2′,7′-dichlorodihydrofluorescein diacetate (H_2_DCFDA, Sigma-Aldrich, St. Louis, MO, USA), a well-established fluorogenic probe for intracellular hydrogen peroxide detection [66]. H_2_DCFDA readily diffuses across the cell membrane and is subsequently hydrolyzed by intracellular esterases to non-fluorescent H_2_DCF. Oxidation of H_2_DCF by ROS generates highly fluorescent 2′,7′-dichlorofluorescein (DCF), which accumulates in the cytosol. DCF exhibits characteristic excitation and emission wavelengths of 498 nm and 522 nm, respectively. The fluorescence intensity of DCF is directly proportional to the concentration of ROS within the cell.

Cells were seeded in black 96-well fluorometric microplates at a density of 10 × 10^3^ cells/mL. After 24 h of cell culture, the cells were treated with the investigated compounds at their IC_50_ concentrations for another 24 h. Following compound incubation, the medium was removed, and the cell monolayers were washed twice with HBSS (140 mM NaCl, 5 mM KCl, 0.8 mM MgCl_2_, 1.8 mM CaCl_2_, 1 mM Na_2_HPO_4_, 10 mM HEPES, and 1% glucose). A volume of 50 µL of H_2_DCFDA solution in HBSS (final concentration 5 μM) was added to each well, and the cells were incubated for 30 min in the dark at 37 °C. After incubation with the H_2_DCFDA, fluorescence measurements were conducted using a Perkin-Elmer LS-5B spectrofluorometer (MA, USA) at the following excitation and fluorescence emission wavelengths: λ_ex_ = 498 nm/λ_em_ = 522 nm. The fluorescence intensity of control cells was arbitrarily assigned a value of 100%. The results were expressed as percentages of control, calculated by comparing the fluorescence intensity ratio of the test samples to controls (100%). The fluorescence of samples was denoted as Δf in relation to the control.

### 3.7. Evaluation of the Role of Reactive Oxygen Species in the Cytotoxic Effects of Flavanone Derivatives

To elucidate the involvement of ROS in the cytotoxic effects of the investigated compounds, cells were pre-treated with the vitamin E (Trolox, Sigma-Aldrich, St. Louis, MO, USA) or N-Acetylcysteine (NAC) antioxidants for 1 h. Cells were seeded into sterile, colorless 96-well microplates at a density of 6–8 × 10^3^ cells/mL, contingent upon the cell line. Following a 24 h culture period, Trolox or NAC was added at a final concentration of 50 μM and 3 mM, respectively. After 1 h of incubation, the antioxidants were removed by washing, and test compounds were administered at appropriate concentrations (10–200 μM) in fresh cell culture medium, followed by a 24 h incubation. The MTT assay was then conducted under identical experimental conditions, as detailed in Section 2.2.

### 3.8. Determination of Glutathione Content in Cancer Cells Using Monochlorobimane

The tripeptide γ-glutamylcysteinylglycine GSH serves a pivotal function in safeguarding cells against the deleterious effects of oxidative stress. Glutathione plays a critical role in antioxidant defense, nutrient metabolism, and a myriad of cellular processes and reactions, including gene expression, DNA and protein synthesis, proliferation, apoptosis, and intracellular signaling. As a potent antioxidant, GSH directly interacts with reactive oxygen and nitrogen species, as well as free radicals. GSH deficiency renders cells vulnerable to oxidative damage. Low GSH levels have been implicated in the pathogenesis of numerous diseases, including cancer. Alterations in the concentration of reduced glutathione may signify disruptions in the redox balance.

The spectrofluorimetric method with monochlorobimane (MCB; Sigma-Aldrich; St. Louis, MO, USA) was used to determine GSH. Monochlorobimane reacts with glutathione in a reaction catalyzed by cellular glutathione S-transferase. The reaction produces a fluorescent bimane-glutathione conjugate (GSH-MCB). A major advantage of MCB is its ability to penetrate the cell membrane, which is why it is used as a probe to determine the amount of GSH in living cells.

Cells were seeded in black 96-well fluorometric microplates at a density of 10 × 10^3^ cells/mL. After 24 h of cell culture, the cells were treated with the investigated compounds at their IC_50_ concentrations for another 24 h. Following compound incubation, the medium was removed, and the cell monolayers were washed twice with HBSS (140 mM NaCl, 5 mM KCl, 0.8 mM MgCl_2_, 1.8 mM CaCl_2_, 1 mM Na_2_HPO_4_, 10 mM HEPES, and 1% glucose). A solution of MCB at a final concentration of 40 μM was added to each well and incubated in a CO_2_ cell culture incubator at 37 °C for 20 min. A 40 mM MCB stock solution was prepared by dissolving 9 mg of the reagent in 1 mL of absolute ethanol. Fluorescence was measured using a Perkin-Elmer LS-5B spectrofluorometer (Waltham, MA, USA) at the following excitation and fluorescence emission wavelengths: λ_ex_ = 380 nm/λ_em_ = 470 nm. After incubation with MCB, the cells were lysed with 50 μL of lysis buffer (10 mM TRIS-HCl; pH 8 containing 0.1% Triton X-100). In order to normalize the number of cells in individual samples, the amount of DNA was determined by adding 50 μL of ethidium bromide (EB, final concentration 0.2 mM) to each well after measuring the MCB fluorescence and measuring the EB fluorescence at an excitation wavelength of λ_ex_ = 530 nm and an emission wavelength of λ_em_ = 590 nm. The results obtained using the standard curve were converted to nM of GSH.

### 3.9. Assessment of Autophagy Induction in Cancer Cells Using the Spectrofluorimetric Method

Autophagy induction in cells treated with the tested drugs was determined by measuring Monodansylcadaverine (MDC; Sigma-Aldrich; St. Louis, MO, USA) fluorescence according to a modified method described by Vázquez [67]. MDC exhibits autofluorescent properties at an excitation wavelength of 365 nm due to the presence of a dansyl group conjugated to cadaverine. In the cell, MDC accumulates in lipid-rich cell membranes. It has been shown that MDC can be used as a dye for detecting autophagy, as it accumulates in autophagolysosomes as a result of interaction with membrane lipids [68].

Cells were seeded in black 96-well fluorometric microplates at a density of 10 × 10^3^ cells/mL. After 24 h of cell culture, the cells were treated with the investigated compounds at their IC_50_ concentrations for another 24 h. Following compound incubation, the medium was removed, and the cell monolayers were washed twice with HBSS (140 mM NaCl, 5 mM KCl, 0.8 mM MgCl_2_, 1.8 mM CaCl_2_, 1 mM Na_2_HPO_4_, 10 mM HEPES, and 1% glucose). A solution of MDC at a final concentration of 50 μM was added to each well and incubated in a CO_2_ cell culture incubator at 37 °C for 15 min. After incubation with MDC, the probe was removed, and the cells were lysed with 50 μL of lysis buffer (10 mM TRIS-HCl; pH 8 containing 0.1% Triton X-100). After lysis, fluorescence was measured at an excitation wavelength of λ_ex_ = 390 nm and an emission wavelength of λ_em_ = 460 nm using a Perkin-Elmer LS-5B spectrofluorometer (Waltham, MA, USA). In order to normalize the number of cells in individual samples, the amount of DNA was determined by adding 50 μL of EB (final concentration 0.2 mM) to each well after measuring the MCB fluorescence and measuring the EB fluorescence at an excitation wavelength of λ_ex_ = 530 nm and an emission wavelength of λ_em_ = 590 nm. The results were calculated as the ratio of MDC fluorescence to EB fluorescence.

### 3.10. Analysis of DNA Damage in Cancer Cells Using the Alkaline Comet Method (Single-Cell Electrophoresis)

Single-cell gel electrophoresis, also known as the comet assay, is a rapid and sensitive method for assessing DNA damage at the single-cell level. Cells are lysed and electrophoresed. Both single-strand and double-strand breaks are detected under alkaline conditions. The released DNA is denatured and moves in the agarose gel toward the anode during electrophoresis. The image, obtained after staining with the fluorescent dye 4′,6-diamidino-2-phenylindole (DAPI), resembles a “comet” with a distinct “head” (intact DNA) and “tail” (damaged DNA fragments). The length of the tail depends on the number and size of the broken DNA strands [69].

Cells (1 × 10^3^) were seeded in 35 mm dishes and cultured in the medium for 24 h to attain the logarithmic growth phase. Following a 24 h culture period, the tested compounds (IC_50_ concentration) were added, and cells were incubated for an additional 12 h. Subsequently, cells were trypsinized and centrifuged for 10 min at 100× *g* at 4 °C. A small amount of PBS and a 0.5% solution of low-melting-point agarose (LMP agarose) were added to the obtained cell pellet and placed on a glass slide covered with two layers of normal-melting-point agarose (NMP agarose). The slides were incubated for 24 h in a lysis buffer (2.5 M NaCl, 100 mM, Na_2_EDTA, 10 mM, Tris, 1% Triton X-100) to release DNA. In the next step, the slides were washed 3 times with a development buffer (1 mM, Na_2_EDTA, 300 mM NaOH) and incubated in the same buffer for 20 min. Then, electrophoretic separation was performed for 20 min (29 V, 300 mA). Quantitative analysis of the DNA comet image of preparations stained with DAPI solution (2 μg/L) was performed using a camera connected to a fluorescence microscope. The comet DNA analysis was performed under a Nikon Eclipse fluorescence microscope (Nikon, Tokyo, Japan) equipped with a 4910 COHU video camera (Cohu, Inc., San Diego, CA, USA) and a computer with Lucia-Comet v. 4.51 software installed (Imaging Laboratory, Prague, Czech Republic). Each time, from each preparation, 100 randomly selected comets were counted. The extent of DNA damage is quantified by measuring the length of the comet tail and the amount of DNA it contains. The Lucia Comet software calculated the percentage of DNA damage based on measurements of color intensity and comet tail length. The intensity of fluorescence was determined by the software, which calculated a series of parameters characterizing the extent of DNA damage for each cell. The percentage of DNA in the comet’s “head” corresponds to genetic material located within the nucleus, while the fluorescent region of the “tail” represents the genetic material that has migrated out of the cell nucleus during electrophoresis.

### 3.11. Cell Cycle Analysis via Flow Cytometry

The most commonly used fluorescent dyes for cell cycle analysis are fluorochromes that penetrate the cell membrane into the interior of permeabilized cells and bind stoichiometrically to DNA via intercalation, e.g., DAPI, propidium iodide (PI). The analysis is performed on cells fixed in ethanol. In this work, the cell cycle analysis was performed using the method described by Darzynkiewicz and Juan [70].

Cells (10 × 10^3^) were seeded in 35 mm dishes and cultured in the medium for 24 h to attain the logarithmic growth phase. Following a 24 h culture period, the tested compounds (IC_50_ concentration) were added, and cells were incubated for an additional 24 h. Subsequently, cells were trypsinized and centrifuged for 10 min at 400× *g* at 4 °C. The cell suspension in 100 μL of PBS was fixed in 1 mL of 70% ethanol (−20 °C) and stored at this temperature until analysis. Immediately before analysis, the ethanol-fixed cell suspension was centrifuged at 10,000 rpm for 10 min at 4 °C. The pellet was washed with cold (4 °C) PBS to remove residual ethanol, and the cell suspension was centrifuged again under the same conditions. A volume of 300 μL of PBS containing 4 mg/mL propidium iodide (final concentration 75 μM) and 1 mg/mL RNAse A (final concentration 20 μg/mL) was added to the cell pellet and incubated for at least 30 min at 37 °C in the dark. Stained cells were analyzed using a flow cytometer (Becton Dickinson, Franklin Lakes, NJ, USA), counting 10,000 cells in each sample. Data were analyzed using FlowJo 10.10 software (FlowJo, Williamson Way Ashland, OR, USA). 

### 3.12. Statistical Significance Analysis

The statistical significance of the results was assessed using the Statistica v. 13.3 (StatSoft Polska, Krakow, Poland). One-way ANOVA analysis of variance and Tukey’s post hoc test for multiple comparisons were used. The significance level of the results was *p* < 0.05. The arithmetic mean and standard deviation were calculated using Microsoft Excel (Microsoft Office 2019, Microsoft Corporation, Redmond, WA, USA). The results of the study were presented in the form of arithmetic mean and standard deviation (SD).

## 4. Conclusions

In this work, the anticancer properties of *3*-benzylideneflavanones/*3*-benzylidenechromanones and their spiropyrazoline analogs condensed with flavanone or chroman-4-one rings were examined in relation to human colon cancer cell lines. The studies constitute the initial phase of evaluating the molecular mechanisms underlying the anticancer activity of the tested derivatives. These compounds were found to exhibit significant cytotoxic and cytostatic effects, primarily through cell cycle arrest in the G2/M phase, leading to cell death. The primary mechanism of action involves the induction of oxidative stress by generating ROS and depleting intracellular GSH.

A critical factor influencing the overall antiproliferative and cytotoxic capacity of the tested flavanone/chromanone derivatives is their pro-oxidant properties and the subsequent oxidative stress generated within cancer cells. The derivatives significantly increased superoxide anion radical levels and moderately elevated hydrogen peroxide levels in cancer cells. Concomitantly, intracellular GSH levels decreased, further contributing to oxidative stress and sensitizing cancer cells to the applied therapy. The induction of oxidative stress was intrinsically linked to genotoxicity and mitochondrial dysfunction, as evidenced by alterations in mitochondrial membrane potential.

A clear correlation between oxidative stress, ROS generation, and the cytotoxic potential of the studied compounds was confirmed by experiments involving the antioxidants NAC and Trolox. Pre-treatment with NAC or Trolox significantly attenuated the cytotoxic effects of the compounds, underscoring the pivotal role of oxidative stress in their anticancer activity.

Collectively, the results of this study suggest that flavanone/chromanone derivatives hold promise as potential anticancer agents. Their ability to induce oxidative stress and disrupt cellular redox balance highlights their potential to overcome drug resistance and enhance the efficacy of conventional chemotherapy. However, the selectivity of the derivatives’ action remains a significant challenge that requires further investigation. A strategy to mitigate systemic cytotoxicity is essential.

Moreover, additional studies are necessary to fully elucidate the molecular mechanisms underlying the anticancer activity of these compounds and to assess their therapeutic potential in vivo. The second phase of the research will focus on a more in-depth analysis of the molecular mechanisms of the anticancer activity of the three flavanone/chromanone derivatives studied. This will involve analyzing the expression of specific proteins involved in apoptosis, autophagy, and cell cycle control (including cyclins). The potential impact of the compounds on key cell fate signaling pathways, such as the mitogen-activated protein kinase (MAPK) pathway, will also be investigated. A crucial aspect of our further research will be to analyze the effects of the flavanone/chromanone derivatives on the activity of nuclear factor erythroid 2-related factor 2 (Nrf2), a transcription factor that regulates cellular antioxidant systems in response to oxidative stress. Nrf2 plays a significant role in chemotherapy and may contribute to cancer development and progression.

## Figures and Tables

**Table 1:** In vitro cytotoxicity activity of flavanone/chromanone derivatives (**1**, **2**, **3**, **3a**, **4,** and **5**) and reference compound cisplatin against various human colon cancer cells and the immortalized human microvascular endothelial cell line HMEC-1 as a model of normal cells. Results are expressed as the mean ± SD of three repeated experiments. IC_50_ values (µM) were calculated at the concentration of a tested compound able to reduce the fraction of surviving cells to 50% versus control probe (non-treated cell).

Cell Line	Cytotoxic Effect of Flavanone/Chromanone Analogs and Reference Compound Cisplatin, IC_50_ (µM)						
	1	2	3	3a	4	5	Cisplatin
HCT 116	10.1 ± 1.3	191.5 ± 3.4	18.6 ± 1.4	74.7 ± 5.2	112.7 ± 5.1	23.7 ± 2.2	8.7 ± 1.2
SW620	8.1 ± 1.7	211.1 ± 2.9	12.4 ± 2.1	91.5 ± 1.9	>250	16.7 ± 3.1	8.1 ± 1.1
LoVo	12.6 ± 1.5	199.7 ± 6.6	23.2 ± 1.4	156.1 ± 6.3	177.5 ± 4.4	19.4 ± 2.1	9 ± 1.2
Caco-2	10.2 ± 1.8	>250	69.9 ± 5.1	97.8 ± 3.2	156.1 ± 5.2	72.1 ± 1.7	5.9 ± 1.4
HT-29	21.1 ± 1.3	>250	116.7 ± 6.1	187.8 ± 3.6	172.4 ± 4.2	31.1 ± 1.4	131 ± 6.6
HMEC-1	21.2 ± 3.1	-	24.1 ± 1.3	-	-	19.7 ± 3.7	9.8 ± 2.6

**Table 2:** In vitro cytotoxicity of flavanone/chromanone derivatives **1**, **3,** and **5** after 1 h of pre-incubation with NAC or Trolox. The cells were incubated with the investigated compounds for 24 h. Results are expressed as the mean ± SD of three repeated experiments. IC_50_ values (µM) indicate the concentration of a compound required to reduce the fraction of surviving cells to 50% compared to the control probe (i.e., an untreated cell).

Cell Line	Cytotoxic Effect of *3*-Benzylideneflavanone/*3*-Benzylidenechromanone Derivatives After 1 h of Incubation with Antioxidants NAC or Trolox (µM)					
	1 + NAC	1 + Trolox	3 + NAC	3 + Trolox	5 +NAC	5 + Trolox
HCT 116	17.1 ± 1.3	9.1 ± 1.4	47.3 ± 6.0	18.4 ± 4.0	23.7 ± 2.1	32.2 ± 2.8
SW620	22.4 ± 2.7	5.2 ± 1.7	24.8 ± 5.7	17.1 ± 6.4	36.1 ± 2.1	27.7 ± 2.8
LoVo	25.0 ± 3.7	13.2 ± 3.1	36.6 ± 4.7	66.9 ± 6.4	30.0 ± 3.2	31.3 ± 4.3
Caco-2	9.3 ± 1.4	12.3 ± 3.4	-	-	-	-
HT-29	24.1 ± 3.1	12.3 ± 2.4	-	-	43.23 ± 2.5	28.4 ± 4.0

## Data Availability

The original contributions presented in the study are included in the article. Further inquiries can be directed to the corresponding authors.
